# A Focused Review on Cognitive Improvement by the Genus *Salvia* L. (Sage)—From Ethnopharmacology to Clinical Evidence

**DOI:** 10.3390/ph16020171

**Published:** 2023-01-23

**Authors:** Abdulselam Ertas, Serkan Yigitkan, Ilkay Erdogan Orhan

**Affiliations:** 1 Department of Analytical Chemistry, Faculty of Pharmacy, Dicle University, Diyarbakir 21200, Türkiye; 2 Department of Pharmacognosy, Faculty of Pharmacy, Dicle University, Diyarbakir 21200, Türkiye; 3 Department of Pharmacognosy, Faculty of Pharmacy, Gazi University, Ankara 06330, Türkiye

**Keywords:** *Salvia*, sage, memory enhancement, ethnopharmacology, clinical study, Alzheimer’s disease, cognitive dysfunction

## Abstract

Ethnopharmacology has been an important starting point in medical and pharmaceutical sciences for discovering drug candidates from natural sources. In this regard, the genus *Salvia* L., commonly known as sage, is one of the best-known medicinal and aromatic plants of the Lamiaceae family; it has been recorded as being used for memory enhancement in European folk medicine. Despite the various uses of sage in folk medicines, the records that have pointed out sage’s memory-enhancing properties have paved the way for the aforementioned effect to be proven on scientific grounds. There are many preclinical studies and excellent reviews referring to the favorable effect of different species of sage against the cognitive dysfunction that is related to Alzheimer’s disease (AD). Hence, the current review discusses clinical studies that provide evidence for the effect of *Salvia* species on cognitive dysfunction. Clinical studies have shown that some *Salvia* species, i.e., hydroalcoholic extracts and essential oils of *S. officinalis* L. and *S. lavandulaefolia* leaves in particular, have been the most prominently effective species in patients with mild to moderate AD, and these species have shown positive effects on the memory of young and healthy people. However, the numbers of subjects in the studies were small, and standardized extracts were not used for the most part. Our review points out to the need for longer-term clinical studies with higher numbers of subjects being administered standardized sage preparations.

## 1. Introduction

Memory deficit, i.e., episodic and spatial memory shortage, thinking disabilities and cognitive dysfunction, are mostly associated with Alzheimer’s disease (AD), a progressive neurological disease commonly seen in elderly populations as well as people with Parkinson’s disease (PD) [1,2,3,4]. Although memory impairment is one of the characteristic symptoms of AD, the pathophysiology of the disease is quite multifarious, which can be explained by two principal hypotheses, i.e., amyloid and cholinergic [5,6]. The amyloid hypothesis is based on the formation of amyloid plaques at the extracellular level and the intracellular buildup of neurofibrillary tangles [7]. Meanwhile, cholinergic hypothesis, the earliest theory, is based on the deficit in acetylcholine (ACh) level, which is a cholinergic neurotransmitter that is hydrolyzed by acetylcholinesterase (AChE) [8,9,10,11,12]. Relevantly, butyrylcholinesterase (BChE), the sister enzyme of AChE of the serine hydrolase class, has broader varieties of substrates compared with AChE that can also inhibit ACh [13]; BChE has been histochemically found to accumulate in *β*-amyloid rich-senile plaques in the brain [14,15,16,17]. At present, only two drug classes, i.e., cholinesterase inhibitors (i.e., tacrine, rivastigmine, donepezil, and galanthamine) and *N*-methyl-D-aspartate (NMDA) antagonists (i.e., memantamine), have been used for the treatment of AD [18,19,20,21,22,23]. On the other hand, a growing number of studies on inhibitors of the secretase family (e.g., β-site APP cleaving enzyme-1, and BACE1), particularly *β*- and *γ*-secretases, may provide additional therapeutic strategies for the future route of AD [24,25,26,27,28]. In addition to cholinergic and amyloid mechanisms underlying AD, the complicated pathology of AD has been also strongly associated with neuroinflammation, oxidative stress, metal accumulation, microglia heterogeneity, sirtuins, mitochondrial dysfunction, etc. [29,30,31,32,33,34,35,36,37,38,39,40,41,42].

On the other hand, monoclonal antibodies such as aducanumab and gantenerumab, which serve as anti-amyloid agents and act through the accumulation of amyloid-*β* peptides in neurons, may be licensed in near future [43,44]; it also should be noted that EMERGE and ENGAGE clinical trials conducted with aducanumab were not found to be satisfactory by the U.S. Food and Drug Administration (FDA) Advisory Committee or the European Medicine Agency (EMA) [45,46,47]. Currently, symptomatic pharmacotherapy is being applied against AD, where a definitive treatment method to completely stop the disease has not yet been found; instead, the treatment is designed to slow its progression. Consequently, a certain need still exists for more effective and safer novel candidates towards AD.

As can be seen, while great efforts are ongoing to find novel drug candidates for AD, herbal medicines may contribute to relevant research that is based on the need for safer and more effective drugs to slow the disease and improve the quality of life of patients. Therefore, plant extracts/natural compounds, medical treatments and preventive interventions that improve the quality of life continue to grow in the world day by day [48,49]. To date, a number of medicinal and aromatic plants have been reported for their positive noteworthy effects on human memory and cognitive activities [50,51]. Unquestionably, the most popular herbal remedy currently used in clinical practice for AD is *Ginkgo biloba* L. leaf extract. Preparations containing *G. biloba* leaf extract standardized over its flavonoids and sesquiterpene lactones (coded EGb 761) has been reported to mainly exert its effect through its vasodilation and antioxidant properties against AD [52,53,54,55].

Without a doubt, the ethnopharmacological data of a specific medicinal use of a plant in folk or traditional medicinal systems have seriously guided research on the topic, and so far, this data has led to the discovery of many clinically available drugs [56,57,58,59]. Furthermore, many studies have pointed out that examining the targeted pharmacological effect of a plant used in folk medicine as the starting grounds for research is a more successful approach in terms of reaching bioactive molecules [60,61,62,63]. Various species of *Salvia* have been reported to be used against symptoms of forgetfulness in European and South African folk medicine, as well as in traditional Chinese medicine (TCM) [64,65,66]. For instance, the potentially beneficial effects of huperzine A, an alkaloid isolated from *Huperzia serrata* (Thunb.) Trevis., a plant used in TCM against memory deficits, have been linked with the alkaloid’s effect on the cholinergic system; huperzine A (shuangyiping in Chinese) was licensed for the treatment of Alzheimer-type dementia in China in 1991, whereas it is traded as a dietary supplement in the rest of the world [67,68,69,70,71]. Similarly, the traditional use of *Salvia officinalis* L. and *S. lavandulaefolia* Vahl. (Spanish sage) in Europe and the use of *S. miltiorrhiza* Schleid. (danshen) for this purpose in TCM has been confirmed [72,73,74,75,76,77].

Based on a retrospective review on the historical role of a number of European plants on the improvement of memory or cognition, *S. officinalis* (sage) along with *Melissa officinalis* L. (lemon balm), another well-known species in the Lamiaceae family, was mentioned for the first time by Perry et al. [64,78,79] for their use against memory deficit. In light of this ethnopharmacological record, Perry’s research group conducted experiments on sage plant to verify its relevant effects using in vitro and in vivo methods, followed by clinical trials [80]. Similarly, *S. officinalis* was shown to display acetylcholine receptor activity by having both nicotinic and muscarinic binding properties [81]. Furthermore, a recent study exhibited that *M. officinalis*, which has the same acetylcholine receptor activity in acute administration in healthy young volunteers, modulated mood and cognitive performance [82]. No side effects or symptoms of toxicity associated with the use of *S. officinalis* have been reported in those particular studies [81,82]. Although a considerable number of medicinal and aromatic plants have been reported to be used against memory impairment in folk medicine, and although some have reported that these plants exhibit memory-enhancing effects, the overwhelming majority of the studies have been at the pre-clinical level. Only a limited number of clinical trials were performed with sage species, which were specifically reviewed a few times in earlier years [83,84,85,86]. The purpose of the present review is to appraise the clinical studies on the memory-improving effects of sage in particular and to update the recent outcomes. Clinical studies and memory-related articles on sage have been searched through various databases, including the Web of Science (WoS), Scopus, Embase, Cochrane Library, Medline, PubMed, and Google Scholar databases using the key words “*Salvia*, *Salvia officinalis*, *Salvia* and Alzheimer’s disease, clinical trials and *Salvia*, *Salvia* and memory, *Salvia* and cognitive dysfunction”. In addition, www.clinicaltrials.gov (accessed on 28 October 2022), the official website of the National Institute of Health (NIH), was also checked for any clinical data on “*Salvia* and Alzheimer’s disease”. In this review, the therapeutic effect of the *Salvia* species in relation to AD has been scrutinized based on clinical evidence acquired from the mentioned databases.

## 2. Clinical Studies on *Salvia* Species against AD 

### 2.1. Salvia officinalis L.

**Figure 1:**
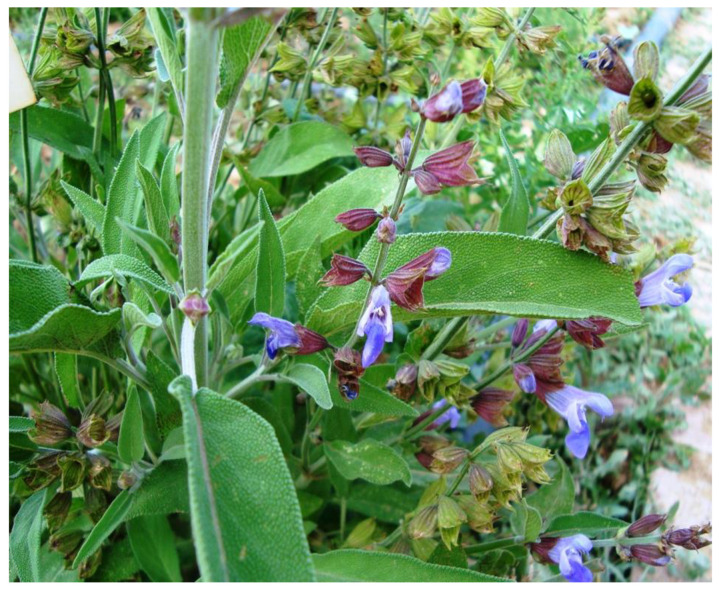
*Salvia officinalis* L. (Lamiaceae) (Photo taken by Ilkay Erdogan Orhan).

Pre-clinical and clinical studies have so far indicated that the extracts and essential oils of *Salvia officinalis* L., which have been used in European folk medicine for memory enhancement, have been favorable for acute memory and attention in healthy young and old participants (Figure 1) [87]. Researchers have largely agreed that the observed effects for the *Salvia* species have resulted from their cholinesterase inhibitory effect [87]. In this context, Akhondzadeh et al. clinically tested *S. officinalis*, which has been used in European folk medicine for centuries, in Alzheimer’s patients that were diagnosed using the National Institute of Neurological and Communicative Disorders and Stroke and Alzheimer’s Disease and Related Disorders Association (NINCDS ⁄ ADRDA) criteria [88]. The stage of the disease in the patients was established through the cognitive subscale scores of the Alzheimer’s Disease Assessment Scale (ADAS-cog) and Clinical Dementia Rating Scale (CDR); based on this, the authors suggested that *S. officinalis* could potentially provide an option for AD therapy based on its in vitro cholinergic binding properties with modulation of mood and its enhancing effect on cognitive performance in humans (Table 1). For instance, a randomized study was conducted by comparing the placebo group in three centers in Tehran (Iran) over a period of four months. The authors evaluated the efficacy and safety of a hydroalcoholic (45%) extract of *S. officinalis* cultivated in Iran at a fixed dose (60 drops/day) in patients with mild to moderate AD, whose ages ranged between 65 and 80 years (average of 71 years). The study revealed that the efficacy of *S. officinalis* extract in the treatment of mild to moderate AD was remarkable. The authors also noted that *S. officinalis* extract may reduce the agitation of patients. Only six patients were recorded to have simple side effects such as vomiting and dizziness. Although it was found to be effective in patients with mild to moderate types of AD, the limitations of the study were concluded as a small number of patients and short follow-up time. On the other hand, no information was available on the phytochemical content of the extract, which is quite indispensable for assessing the pharmacological effect as well as the rational dosing and quality control of herbal medicines (phytotherapeutics) [89,90,91,92].

In another double-blind, placebo-controlled, and crossover study using ethanol extract (53%) prepared from the dried sage leaves of *S. officinalis*, 30 healthy participants (average age: 24.4 years; 17 males and 13 females) attended the laboratory on 3 separate days (7 days apart as the wash-out period) and were administered with doses of 300 and 600 mg of the extract in opaque capsules each time in a balanced mode [87]. Daily mood was assessed 1 h before and 4 h after. The mental state assessment of participants was performed by a completion of Bond–Lader mood scales and the State-Trait Anxiety Inventory (STAI) tests before and 20 min after a performance of a Defined Intensity Stress Simulator (DIS) computerized multitasking application. In the study, the extract inhibited AChE (IC_50_ of 0.365 mg/mL) in vitro and, to a greater extent, BChE (IC_50_ of 0.054 mg/mL), in a dose-dependent manner. Post-dose, both doses of sage in the absence of stressors (i.e., pre-DISS mood scores) exhibited that a low dose reduced anxiety, whereas a high dose increased vigilance, calmness and happiness, resulting in a better rating of mood on the opening Bond–Lader visual analogue mood scales. However, the reduced anxiety effect following the lower dose was abolished by performing the DISS test, and the same dose was also associated with decreased alertness during performance. Eventually, the results obtained from this study confirmed previous observations of the inhibitory properties of *S. officinalis* on cholinesterase and improved mood and cognitive performance after a single dose and acute administration in healthy young participants [87]. The study can be criticized for its small number of human subjects with non-AD and its use of a non-standardized extract of *S. officinalis*, which are shortcomings in the rational evaluation of outcomes of the study.

The same test system was performed in a similar study that recruited 20 healthy participants with a mean age of 72.95 years. The findings pointed out to the fact that the encapsulated standardized ethanolic extract (70%) of the dried leaves of *S. officinalis* extract demonstrated significant improvements in the quality of memory scores, including word presentation, immediate and delayed word recall, simple reaction time, picture presentation, choice reaction time, digit vigilance tasks, spatial and numeric working memory, and delayed word and picture recognition [93]. In addition, the study found that although no effect was observed for subjective mood, there was a noteworthy enhancement in the accuracy of the attention factor. This study investigated the acute effects of standard *S. officinalis* extract on cognitive performance in older adults using a double-blind, placebo-controlled, randomized, five-period, balanced, and crossover model. Participants were given four doses of *S. officinalis* extract (1332, 666, 333, and 167 mg) and placebo with a 7-day wash-out period between each visit. The evaluation was performed with a CDR computerized evaluation battery. Further assessment was performed immediately after baseline assessment 1, 2.5, 4, and 6 h after treatment on study days. The results showed that compared with the placebo condition, a 333 mg dose led to a substantial enhancement in secondary memory performance at the tested times. Secondary memory scores in the extract group were positively influenced in terms single task, normal and delayed word recognition, immediate word recall, accuracy of attention, and working memory tasks, whereas there was no significant improvement in the speed of memory and attention. The authors stated that the standardized *S. officinalis* extract exerted a significant acute enhancement in memory, especially influencing secondary memory factors. However, the use of a limited number of human subjects in the study and the lack of clarity on the basis of standardization can be commented as weaknesses of this clinical trial.

In one of the clinical trials registered at www.clinicaltrials.gov conducted by the Oregon Health and Science University in collaboration with the National Center for Complementary and Integrative Health (NCCIH) (ClinicalTrials.gov identifier/NCT number: NCT00110552), the extract of *S. officinalis* was tested in capsule form on the cognitive function of patients (n: 40) from both sexes aged 50–90 years with mild AD, which started in 2005 and completed in 2014 as the phase 1 study. The interventional trial lasted for 6 weeks in a random design with parallel assignment. Electroencephalogram (EEG) and electrocardiogram (ECG) measurements were taken from the participants at each visit. However, the final results have not yet been posted. 

**Table 1:** Clinical studies conducted on several *Salvia* species.

*Salvia* Species	Preparation Used	Number of Participants	Age Range of Participants	Control Group	Tests Applied	References
*S. rosmarinus*	Hydrolat extract mixture	80	Mean, 23	Plain mineral water	Computerized Mental Performance Assessment System	[50]
*S. rosmarinus*	Dried leaf	28	65–90	Placebo	Cognitive Drug Research	[94]
*S. officinalis*	Dried leaf	30	Young volunteers	Placebo	Defined Intensity Stressor Simulation, Bond–Lader mood scales, State-Trait Anxiety Inventory	[87]
*S. officinalis*	Leaf extract (45% ethanol)	103	65–80	Placebo	Alzheimer’s Disease Assessment Scale, Clinical Dementia Rating Scale	[88]
*S. lavandulaefolia*	Essential oil	44	18–37	Placebo	Cognitive Drug Research computerized test battery	[74]
*S. lavandulaefolia*	Essential oil	24	18–37	Sunflower oil	Cognitive Drug Research computerized test battery	[95]
*S. officinalis*	Leaf extract (70% ethanol)	20	65–90	Placebo	Cognitive Drug Research test	[93]
*S. officinalis*, *S. lavandulaefolia*	Aroma	135	Mean, 22	No aroma	Cognitive Drug Research system Bond–Lader mood scales	[96]
*S. lavandulaefolia*	Essential oil and sunflower oil mixture	11	76–95	-	Cognitive Drug Research computerized cognitive assessment	[83]
*S. lavandulaefolia*	Essential oil	36	Mean, 24	Placebo	Computerized Mental Performance Assessment System	[97]
Cognivia™	*S. officinalis* aqueous extract and *S. lavandulaefolia* essential oil mixture	94	30–60	Placebo	Computerized Mental Performance Assessment System	[98]
*S. officinalis*, *R. officinalis*, *M. officinalis*	Mixture of ethanol (45%) extracts	44	Mean, 61	Placebo	Immediate word recall	[99]
NaO Li Su	Mixture of bee pollen, radix polygoni multiflore, semen ziziphi spinosae, radix salviae multiorhizae, fructus schisandrae, and fructus ligustris lucidae drogs	100	50–69	Placebo	Wechsler Adult Intelligence Scale, Wechsler Memory Scale	[100]
Fufangdanshen	Mixture of *Salvia miltiorrhiza*, *Panax notoginseng*, and *Borneolum syntheticum*	231	45–80	Placebo	Alzheimer’s Disease Assessment Scale, cognitive subscale	[101]
*Beta vulgaris*, *S. officinalis*, *Panax* sp.	Mixture of blueberry, apple, and coffee berry extracts	32	18–35	Placebo	Computerized Mental Performance Assessment System, Cognitive Demand Battery	[102]
*S. divinorum*	Salvinorin A	4	23–35	Placebo	Hallucinogen Rating Scale, Mysticism Scale	[103]
*S. divinorum*	Salvinorin A	32	25–65	Placebo	Hallucinogen Rating Scale	[104]
*S. divinorum*	Dried leaf and extracts	47	19–43	-	Hallucinogen Rating Scale	[105]
*S. divinorum*	Salvinorin A	8	21–35	Placebo	Hallucinogen Rating Scale, Pharmacological Class Questionnaire	[106]

### 2.2. Salvia lavandulaefolia Vahl.

**Figure 2:**
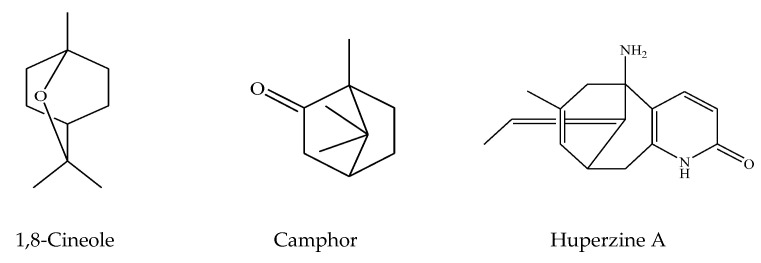

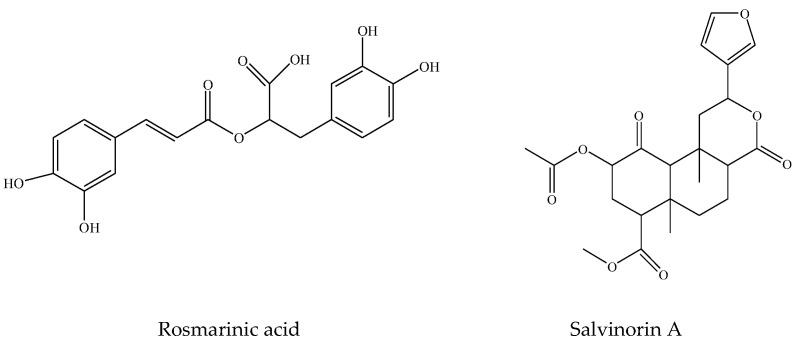
Promising compounds detected in or isolated from *Salvia* species against AD.

Likewise, Spanish sage (*S. lavandulaefolia* Vahl.) has been reported to improve memory and attention in young volunteers [74]. In particular, the cholinergic effects of *S. lavandulaefolia* on memory and cognitive capacity were shown to be mainly due to 1,8-cineole, a monoterpene-derivative compound that can easily cross the blood–brain barrier (Figure 2) [107,108,109]. Sage (*Salvia*) species have a long-standing reputation in British plant encyclopedias as a memory-enhancing plant, although there is a little evidence of their effectiveness through systematic research. Based on its known pharmacokinetic and binding properties, it was assumed that an acute administration of *S. lavandulaefolia* would strengthen memory in young adult volunteers [74]. Two clinical experiments used a placebo-controlled, double-blind, balanced, and crossover methodology. In the placebo-based trial 1, 20 of the participants were given 50, 100, and 150 mL of *S. lavandulaefolia* essential oil, while 24 participants were administrated 25 and 50 mL of *S. lavandulaefolia* essential oil versus placebo in trial 2. The assessment was made using a CDR computerized test battery, conducted before treatment as well as 1, 2.5, 4, and 6 h following the treatment. The primary outcome criteria were determined as the instant and delayed word recall. A 50 mL dose of *S. lavandulaefolia* essential oil was found to significantly improve instant word recall in both studies. These results represent the first systematic evidence that *S. lavandulaefolia* is capable of acute modulation of cognition in healthy young adults.

In another clinical study by Tildesley et al. on the species *S. lavandulaefolia* gave 24 participants a placebo (sunflower oil) or capsule (25 or 50 mL of essential oil) [95]. The effects were observed 1, 2.5, 4, and 6 hours after the capsule administration of each participant. The results at both dosages of *S. lavandulaefolia* essential oil showed significant improvements, when compared with the placebo group. Especially at the 1 h administration period, the lower dosage group caused a noteworthy improvement in secondary memory factors. In addition, different and important developments were recorded in the “content”, “calm”, and “vigilant” aspects of mood, the results of which were evaluated using the Bond–Lader scale.

*S. lavandulaefolia* extracts and its components are known to have antioxidant, anticholinesterase, estrogenic, CNS depressant (sedative), and anti-inflammatory effects, which are currently relevant to the treatment of AD [83]. In a study conducted with 11 healthy volunteers, the application of *S. lavandulaefolia* essential oil had significant effects on cognition. In an open-label pilot study using *S. lavandulaefolia* essential oil that was orally administered to participants, a significant increase in diastolic and systolic blood pressure was observed in two patients with AD. However, this was primarily judged to be due to pre-existing hypertension, and no other vital signs or abnormalities in blood samples and blood pressure were found during the trial period. Statistically significant results were acquired from the reduction of neuropsychiatric symptoms and improvement in attention between the baseline and 6-week treatment [83].

*S. lavandulaefolia* extracts, which are rich in monoterpenes such as 1,8 cineole, were previously known to inhibit cholinesterase enzymes and have positive effects on cognitive function. In line with these data, the essential oil content of *S. lavandulaefolia* was determined, and in vitro cholinesterase inhibitions assays were carried out. At the same time, in parallel with the in vitro studies, the effects of *S. lavandulaefolia* essential oil administered in a single dose on cognitive performance and mood were evaluated in a placebo-controlled, double-blind and crossover study [97]. In the study, 36 healthy subjects were given capsules containing 50 µL of *S. lavandulaefolia* essential oil or a placebo crosswise 7 days apart. The subjects’ cognitive function, rated through a series of computational memory and attention tasks, were assessed using the “Cognitive Demand Battery” prior to treatment, as well as 1 h and 4 h post-dose. According to the results of the chemical analysis of the essential oil used, the oil mostly consisted of monoterpenes. In addition, it was determined to strongly inhibit human AChE. Oral consumption of the essential oil, especially after 1 h, resulted in an increased performance of secondary memory and attention tasks. In addition, it led to a decrease in mental fatigue and increase in alertness, which was more pronounced 4 h after the dose. The results of this study extended previous observations of sage extracts with AChE inhibitory potential regarding improved cognitive performance and mood. It was also noted that the ability of extracts of species such as *S. lavandulaefolia* having well-tolerated monoterpenoids should be further studied for their ability to beneficially modulate cholinergic function and cognitive performance.

Moss et al. examined the potential of using aromas of essential oils of some *Salvia* species to affect cognition and mood in healthy adults [96]. They reported that orally administered *S. officinalis* and *S. lavandulaefolia* have the ability to modulate cognition and mood. The active compounds in herbal products can also be found in aroma and, therefore, may show similar effects. In a study based on an independent group design, the aroma of *S. officinalis* and *S. lavandulaefolia* was administered to 135 healthy adult volunteers that took part in the study. The cognitive performance was assessed through the CDR system and Bond–Lader mood scale to measure participants’ mood in three dimensions before and after cognitive tasks. The results revealed that the *S. officinalis* flavor group performed significantly better than the control group. The alert mood measure revealed significant differences between both the flavors and the control condition. It has been shown that the aroma results of *Salvia* species showed similar results to oral use, but do not fully correspond to each other. In addition, objective and subjective interesting differences were detected between both uses.

It is mentioned in the literature that the consumption of various extracts and dry parts of European and Spanish sage species (e.g., *S. officinalis* and *S. lavandulaefolia*) demonstrated positive psychoactive effects with respect to cognitive activity. It should be noted that despite the similarity in their chemical content, they slightly differed in their chemical compounds. For example, while these two sage species contained 1,8-cineol in similar proportions, their camphor ratios (33.6% and 15.5% camphor, respectively) were quite different [110,111]. Regarding their phenolic compositions, *S. officinalis* synthesizes salvianolic acid derivatives that originate from rosmarinic acid, but *S. lavandulaefolia* does not synthesize or synthesizes less (Figure 2). These compounds have already been reported to display positive effects on alertness and memory in both young and older adults [87,93]. Moreover, monoterpene compounds found in *S. lavandulaefolia* possessed similar properties [74]. Recent studies have confirmed the psychoactive properties of *S. lavandulaefolia* in healthy young adults who consumed its essential oils, which are abundant in monoterpene derivatives. Specifically, this oil containing a high concentration of 1,8-cineol was found to exhibit a particularly potent AChE inhibitory profile. A single dose of this essential oil has been found to improve memory and attention task performance, increase alertness and reduce mental fatigue during long-term performance of difficult tasks [95]. In light of these findings, controlled trials in randomized human groups appear to show strong and consistent acute cognitive effects when administered as a single species (*S. lavandulaefolia* or *S. officinalis*). However, it is not known yet whether chronic effects are common or if they even occur. In addition, it is not known if the synergistic mechanisms of action of these effects can produce completely different effects when the species are applied together. This situation was partially resolved in a recent study by Dinel et al. (2020). In this study, Cognivia™, an herbal preparation obtained from *S. lavandulaefolia* essential oil and *S. officinalis* aqueous ethanol leaf extract was studied in a rodent model [112]. After the administration of Cognivia™ obtained from two sage mixtures, the histological and biochemical parameters were determined in rodents. The outcomes revealed that Cognivia™ stimulated the expression of calcium/calmodulin-dependent protein kinase II (CaMKII), a proposed mechanism for regulating biochemical neuronal processes. Thus, it was disclosed that this herbal product supports learning, working memory, and interpretation facilities [113,114].

In this context, it can be said that sage (*Salvia*) species contain a series of terpenes and phenolic compounds that interact thorough the mechanisms related to brain function and improve the properties of cognitive performance [98]. However, previous studies in humans have examined these phytochemicals by only following their acute consumption. Of relevance, a study in rodents exhibited improved cognitive outcomes after 2 weeks of consumption of Cognivia™, a proprietary extract of both *S. officinalis* polyphenols and *S. lavandulaefolia* terpenoids [112]. This suggests that a combination of phytochemicals from sage might be more effective when used over a longer period of time. In the study of Wightman et al., the effect of these two sage combinations on the cognitive functions of people with their acute and chronic consequences was investigated. The participants (n: 94; 25 males and 69 females, 30–60 years of age) were placed in a randomized, double-blind, placebo-controlled, and parallel group design with a 600 mg dose of sage combination or placebo acutely administered and analyzed at 120 and 240 min following 29 days of supplementation; a comprehensive set of cognitions was evaluated [98].

In conclusion, sage consumption of Cognivia™ in healthy adult humans has been found to have a consistent and significant benefit on the accuracy of working memory and performance of cognitive domains. It was found that this significant activity was observed in both acute (only 2 h after consumption) and chronic individuals (29 days after administration). The authors also noted that there was an increase in the product’s efficacy over the course of administration and, when considering combination of the two strains together, the authors noted that future trials should focus on resolving the working and spatial memory effects of this intervention in humans over a long period of time, perhaps over several months [98].

It is undisputed that the need for therapeutic strategies to counter the expanding elderly populations around the world and the cognitive decline in dementia seems more important these days. It is reported that one person in every 67 s is diagnosed with AD in the United States [115]. In addition, recent findings in Europe propose that the incidence may decrease due to improved brain health; furthermore, specific anti-Alzheimer’s therapy should also combine preventive lifestyle interventions that target general brain health. In addition, clinical trials are suggested to be the key for finding effective treatments and preventive strategies [116]. Since the discovery of sage species (*S. lavandulaefolia* and *S. officinalis*) use for the treatment of dementia [78], several traditional medicinal drugs have been shown to improve cognitive functions including memory [80] along with actions associated with improving brain health, such as anti-inflammatory, antioxidant, and neuroprotection. 

### 2.3. Salvia rosmarinus Schleid.

A number of studies investigating the potential of rosemary (*Rosmarinus officinalis* L.; syn. *Salvia rosmarinus* Schleid., http://www.theplantlist.org/tpl1.1/record/kew-179873, accessed on 2 November 2022) extracts and components to provide anti-amyloid [117], neuroprotective [118] and antidepressant [119] effects through monoaminergic neurotransmission and antioxidant activity are available in the literature. However, there are only two human trials in the literature where rosemary extracts were orally used for study on human cognition. A small study comparing 1600 mg of dried rosemary plant with a placebo displayed a moderate effect in favor of the rosemary group [94]. In another study, a balanced cross-design study recruiting older participants investigated the possible different effects of a dose of rosemary on a range of cognitive measures [100]. In the study, it was found that although a dose of 750 mg improved performance in memory processing speed, the highest dose (6000 mg) caused a decrease in performance. Such an “inverse U”-shaped curve is characteristic of pharmacological cognitive enhancers, which was noted to be associated with the absorption of orally administered 1,8-cineole in an in vivo study [50,109].

In a study by Moss et al. [50], the effect of consuming commercially available water containing rosemary extract and hydrolyte was investigated through the dimensions of cognitive function, mood, and cerebrovascular response as dependent variables, which were measured by near-infrared spectroscopy. In the study, 80 healthy adults were randomly assigned to consume 250 mL of rosemary water or plain mineral water. Then, the participants completed a series of computerized cognitive tasks followed by subjective measures of wakefulness and fatigue. The total, oxygenated and deoxygenated hemoglobin levels were monitored by near-infrared (IR) spectroscopy at baseline and throughout the cognitive test procedure. The analysis of the data, in line with studies in the literature conducted by inhalation of rosemary essential oil and aroma, determined that rosemary water has a number of statistically significant, small, favorable effects on cognition. Of particular interest here, cerebrovascular effects were noted for oxygen–deoxygenated hemoglobin levels during the cognitive task performance, which were significantly higher when the rosemary water was used. These new findings suggested that oxygen extraction is facilitated at times of cognitive demand. Taken together, the data pointed out to potential beneficial properties of acute consumption of rosemary water. In the study, the findings were discussed in terms of assumed metabolic and cholinergic mechanisms.

### 2.4. Salvia miltiorrhiza Schleid.

In addition to *S. officinalis* and *S. lavandulaefolia*, a couple of clinical studies on *S. miltiorrhiza* are available. The mixture known as “NaO Li Su”, consisting of Radix Polygoni multiflore, Fructus Ligustris lucidae, Radix Salviae miltiorrhizae, Fructus Schisandrae, bee pollen, and Semen Ziziphi spinosae, is used in traditional Chinese medicine (TCM) [120]. This mixture has been used in TCM as a memory enhancer for a long time. In a clinical study with this mixture, Iversen et al. evaluated 100 elderly Danish volunteers suffering from memory impairment by a series of psychological and biochemical tests using a double-blind, placebo-controlled, and crossover 3-month treatment period procedure. Red blood cell counts and serum creatinine levels rose, but active treatment failed to have the expected impact on cognitive skills. Wechsler’s Memory Scale scores and changes in the quantity of these red blood cells were found to be significantly positively correlated.

Following AD, vascular dementia (VaD) is the second most prevalent dementia subtype worldwide; there is currently no medication authorized to treat VaD patients. The Chinese Food and Drug Administration (CFDA) authorized the traditional herbal medicine known as Fufangdanshen tablets (FFDS) in 2008, which is composed of Chinese traditional herbs, e.g., *S. miltiorrhiza* and *Panax notoginseng* (Burkill) F.H.Chen along with synthetic borneol; FFDS tablets are used to treat patients with VaD. Chemical analyses on this preparation identified the existence of borneol, *Panax* notoginsenosides, salvianolic acid, ginsenosides Rg1 and Rb1, and tanshinone as major components. Tanshinone has been shown in preclinical tests to alleviate A1-40-induced memory and learning deficits in rat models of AD [120,121]. Additionally, tanshinone decreased harmful free radicals, prevented oxidative damage and inhibited the expression of AD-induced inducible nitric oxide synthase (iNOS) and matrix metalloproteinase II (MMP-2) [122]. Salvinolic acid, which is present in FFDS tablets, was shown to have inhibitory effects on anti-cerebral ischemia effects and glutamate release, whereas borneol has been reported to promote blood–brain barrier permeability [101,123].

Tian et al. conducted a 24-week randomized, double-blind, parallel, and multicenter phase 1 study to further evaluate the effectiveness, tolerability and safety of FFDS tablets in the treatment of mild to moderate VaD, denoting that there are methodological weaknesses in medical care using FFDS tablets [124]. The patients (n: 240), who were diagnosed with mild to moderate VaD, participated in a randomized experiment, where they were randomly assigned to receive either three FFDS tablets or a placebo tablet three times per day for 24 weeks. Appropriate patients were chosen after a 2-week break-in period, and after 12 weeks, individuals were followed up on. The Clinician Interview-Based Impression of Change (CIBIC-plus) and ADAS-cog tests were chosen as the main indicators of success; the Mini-Mental State Examination (MMSE) results, activities of daily living (ADL) and adverse reactions were also recorded as secondary indicators of efficacy. In the end of the study, only 11 patients reported side effects such as allergy, hypokalemia, fatigue, bloating, thrombocytopenia, sinus bradycardia, and tuberculosis hemoptysis. The trial is still ongoing.

### 2.5. Salvia divinorum Epling and Jativa

The main psychoactive element of *S. divinorum*, a member of the Lamiaceae family that has historically been utilized in ethnopharmacological applications, is salvinorin A, a neoclerodane type of diterpene. It is a *κ*-opioid agonist and distinct hallucinogenic substance with growing popularity as a recreational substance in recent years [125,126]. Studies conducted on animals have defined distinct pharmacological, behavioral and unique effects of salvinorin A and *S. divinorum* (Figure 2) [127]. Salvinorin A and similar substances have been used in numerous research studies to investigate *κ*-opioid pathways in drug addiction cases, neurological illnesses (e.g., AD) and mental disorders [103,126,127]. Nonetheless, there are also a number of human and laboratory studies that may be found in the literature [104,128,129,130]. 

Most of the knowledge concerning *S. divinorum*’s impact on people has come from qualitative user interviews and survey studies. In comparison to other medications or techniques that cause changes in consciousness, the survey respondents generally concurred that the effects of *S. divinorum* were “intense” and “unique” [131,132,133,134]. There may be some overlap with the subjective outcomes of traditional serotonergic hallucinogens based on the rise in Hallucinogen Rating Scale (HRS) scores [105,131]. However, 25% of study participants claimed that the mentioned effect of *S. divinorum* was comparable to conventional hallucinogens [131,132]. The findings addressing *S. divinorum*’s resemblance to cannabis are more nuanced. Beyond the acute effects, less evidence exists on the promoting effect of *S. divinorum* in psychological dependence or psychiatric disorders [134]. The literature contains conflicting accounts on “positive effects like those of antidepressants” [132] and “negative effects including mental blurring” [133]. In actuality, the majority of those surveyed said that they used *S. divinorum* for entertainment or enjoyment, while several also claimed that they used it for spiritual reasons [131,132,133]. 

When examining clinical studies using *S. divinorum*, Karam et al. conducted an investigation on the prevalence and effect of *S. divinorum* use in patients who applied for the detoxification of illicit drug use [106]. In this study, which involved the detoxification of illicit drug abuses of 47 drug users in a psychiatric hospital in Lebanon, 67% of the participants reported using *S. divinorum*. It is known that the herb has a rapid effect 15 min following its use. In terms of cognition and somesthesia sub-dimensions of HRS, no significant difference was found between those who did not use sage as well as those who used sage. While the subscales of concentration and willpower were higher in those who did not use sage, the perception score was higher in sage users. The use of *S. divinorum* has been reported to cause perceptual alterations and hallucinogenic effects.

In another double-blind and placebo-controlled study (n: 8) by Johnson et al. [104], the effect of the one-month use of salvinorin A on four volunteers was investigated. In this study comparing salvinorin A with classical hallucinogens, a comprehensive evaluation was carried out to identify the unique and overlapping effects of the molecule. Conclusions were drawn on the tolerability, safety and time course of salvinorin A inhaled by four subjects at different doses, ranging from low (0.375 μg/kg) to high (21 μg/kg) doses. These results demonstrated the safety of the inhalation procedure of salvinorin A with consistent and dose-dependent effects on participants’ drug potency ratings. It was determined that the inhalation procedure of salvinorin A showed dose-related increases in the Mystical Type Subjective Effect Scale and HRS scores, which were shown to be sensitive to psilocybin and phenomenologically overlapped with classical hallucinogens. In addition, subjects using salvinorin A noted that this active ingredient elicits unique effects that are uncommon to other hallucinogenic drugs.

In the study by Addy [128], 32 volunteers aged 25–65 years inhaled salvinorin A in a double-blind and placebo-controlled study in hallucinogen-experienced adults. The research evaluated the behavior of the participants during the sessions. Participants completed HRS evaluations, which assessed inebriation immediately after each session. Eight-week follow-up data of subjects using salvinorin A indicated a mix of both negative effects such as inattention, fatigue and headaches as well as positive effects such as aesthetic sensitivity, empathy and positive mood, which lasted more than 24 h after inhalation. Participants stated that cannabis (10%), dissociative (7%) and classical hallucinogens (13% LSD, 10% psilocybin) had less effect than salvinorin A (43%) on dreaming.

Finally, a double-blind, placebo-controlled study by MacLean et al. evaluated the dose-dependent effects of inhaled salvinorin A in individuals with a history of hallucinogen use [135]. In a study of eight healthy adults, subjects using hallucinogens progressively inhaled up to 16 doses of salvinorin A (0.375–21 μg/kg). After administration, the physiological, behavioral, and subjective effects of salvinorin A inhalation on subjects (every 2 min for 60 min) were evaluated. At the end of the sessions, individual qualitative effects were retrospectively appraised through questionnaires. Questionnaires assessing qualitative effects (e.g., Pharmacological Class Questionnaire, HRS) disclosed that the effect of salvinorin A on some subjects overlapped with those of classical serotonergic-mediated hallucinogens. In addition, salvinorin A exhibited dose-related dissociative effects and impaired recognition/remembering memory. In addition, the use of salvinorin A has been reported by subjects to be qualitatively different from other classical hallucinogens. It is also said that salvinorin A elicits a unique profile of cognitive and subjective effects, including strong reactions and amnesia that partially overlap with classical hallucinogenic effects. 

### 2.6. Salvia Species in Combinations

Clinical studies have so far supported the cognitive-enhancing effects of the extracts of three European Lamiaceae herbs, i.e., (*Salvia*, *Melissa*, and *Rosmarinus*) [74,93,99,100]. In this context, Perry et al. reported that a combination of lemon balm, rosemary, and sage (*M. officinalis*, *R. officinalis*, and *S. officinalis*; SRM) species, which are widely used in folk medicine in Europe, exhibited a positive impact on memory and brain function, especially in healthy individuals, where the effects were clinically evaluated using verbal recall [102]. In this regard, a double-blind, randomized, and placebo-controlled pilot study was conducted with 44 healthy volunteers randomized to a placebo group. In the study, the chemical content of the ethanol extract of SRM as a natural product mixture was determined using the high-resolution liquid chromatography–ultraviolet-mass spectrometry/ mass spectrometry (LC-UV-MS/MS) technique, and the product was given to the human subjects for 2 weeks. Immediate and delayed word recall techniques were used to assess memory after the ingestion of either SRM ethanol extract or placebo. In addition, the same practice was carried out on subjects divided into younger and older subgroups. In this pilot study, oral ingestion of SRM ethanol extract at a selected dose and duration of administration was found to be more effective on verbal episodic memory than placebo in healthy subjects under 63 years of age. It was emphasized that the short- and long-term consumption of SRM extract as an adjunct therapy for patients with AD and aging population should be investigated in further detail.

Jackson et al. investigated the effects of the natural products obtained from the combination of ginseng, beetroot, and sage (*S. officinalis*) extracts and phenolic-rich apple, blueberry, and coffee fruit extracts on human cognition [136]. A placebo-controlled, double-blind, randomized, and crossover designed study evaluated the effect of sage, ginseng, and beet beverages on 32 healthy adult individuals aged 18–35 years. In addition, each of the beverages contained a phenolic-rich extract from the fruits of coffee (440 mg chlorogenic acid), blueberry (300 mg anthocyanin), or apple (234 mg flavanols). Cognitive and psychological parameters of the subjects were assessed before drinking and 60, 180, and 360 min after drinking each beverage. The data acquired indicated that the apple and coffee beverages demonstrated strong effects on the subjects’ mood and cognition, while the blueberry extract beverage had less of an effect.

A registered clinical trial (ClinicalTrials.gov identifier: NCT03221894) with a combination granule formulation called Grape^®^ (Beijing Tcmages Pharmaceutical Co., Ltd.), consisting of *Gastrodia elata* (tian ma, 10 g/d), *Acorus tatarinowii* (shi cangpu, 10 g/d), *Epimedium brevicornu* (yin yanghuo, 10 g/d), ginseng (ren shen, 10 g/d), *Polygala tenuifolia* (yuan zhi, 10 g/d), *S. miltiorrhiza* (dan shen, 10 g/d), *Cistanche deserticola* (rou congrong, 10 g/d), *Curcuma aromatica* (yu jin, 10 g/d), *Angelica sinensis* (tian ma, 10 g/d), *Cornus officinalis* (shan zhuyu, 10 g/d), berberine (huang lian, 10 g/d), and *Rehmannia glutinosa* (di huang, 30 g/d), was performed in 120 moderate and severe AD patients (aged 50–85 years from both sexes); the study was conducted under the patronage of Dongzhimen Hospital (Beijing) in collaboration with Peking University Third Hospital, Beijing Hospital, and Chinese PLA General Hospital from 2017 to 2021. The patients were monitored using the Mini-Mental State Examination (MMSE), activities of daily living (ADLs), Neuropsychiatric Inventory (NPI), and CDR scores during the 12 months. Nevertheless, the final data has not yet been posted.

## 3. Conclusion and Remarks

A number of species of the genus *Salvia* L. have been recorded in folk medicine for use against memory deficits. This folkloric/ethnopharmacological registry has paved the way for a significant number of pre-clinical and clinical studies with essential oils and extracts of various *Salvia* species. As summarized in this review, the prominent species in pre-clinical and clinical studies were *S. officinalis* and *S. lavandulaefolia*. A few clinical trials have also been conducted on *S. miltiorrhiza*, which is used in Chinese traditional medicine for forgetfulness. Neurological scores obtained in clinical studies with sage, which is the subject of our review, indicated the positive effects of sage leaf extracts and essential oil against AD, especially in the acute period. Although it has been reported that sage leaf extracts and essential oils provide a medical improvement in the relief of AD and related symptoms, important deficiencies have been observed, especially in clinical studies. At the beginning of these shortcomings, it can be criticized that small numbers of patients were used in the clinical trials. Generally, the clinical studies have been conducted with under 300 patients. Another disadvantage of the studies is that the sage leaf extracts used were not standardized, as standardization required by international or national pharmacopoeias is vital for the quality, safety, and efficacy required of a therapeutic herbal agent, especially for modern phytotherapeutic agents. Moreover, standardization is an essential criterion for the adjustment of therapeutic dose in herbal medicines to establish their indication. However, no pharmacopoeia standardization has been reported in the extracts used in studies examining sage. Another weak point of the clinical studies conducted so far on sage is the lack of long-term (chronic) studies. Therefore, despite the promising therapeutic potential of sage towards AD, there is still a need for more comprehensive, longer-term, and better-planned clinical studies with standardized sage extracts and essential oils of pharmaceutical quality.

## Data Availability

Data will be made available upon request.
